# Eye-Tracking in Assessment of the Mental Workload of Harvester Operators

**DOI:** 10.3390/ijerph19095241

**Published:** 2022-04-26

**Authors:** Bartłomiej Naskrent, Witold Grzywiński, Krzysztof Polowy, Arkadiusz Tomczak, Tomasz Jelonek

**Affiliations:** Faculty of Forestry and Wood Technology, Poznań University of Life Sciences, Wojska Polskiego 28, 60-637 Poznań, Poland; bartlomiej.naskrent@up.poznan.pl (B.N.); krzysztof.polowy@up.poznan.pl (K.P.); arkadiusz.tomczak@up.poznan.pl (A.T.); tomasz.jelonek@up.poznan.pl (T.J.)

**Keywords:** eye-tracking, harvester operator, mental workload, timber harvesting

## Abstract

Harvesting large quantities of timber requires the use of various technical means, including harvesters. The introduction of machine logging has greatly improved safety and reduced accident rates but has also resulted in the risk of musculoskeletal disorders and increased psychological strain. The aim of this study was to determine the level of the mental workload of harvester operators in wind-damaged stands, during daytime and nighttime clearfelling, and during late thinning using the technique of eye-tracking (analysis of saccades and pupil dilation). The highest number of saccades for both felling and processing operations was recorded during daytime and nighttime clearcutting, while the lowest number was recorded in late thinning. For both operations, the highest mean saccade duration was found in late thinning (felling 38.7 ms, processing 36.0 ms) and the lowest in nighttime cutting (felling 33.1 ms, processing 35.5 ms). The highest frequency of saccades in both operations was recorded in clearcut areas during both daytime and nighttime operations. The largest mean pupil diameters during saccades were recorded in night clearfelling plots (felling 5.57 mm, processing 5.52 mm), while the smallest were recorded in plots with windbreaks (felling 2.91 mm, processing 2.89 mm). Comparison of the number, duration, frequency, and time proportion of saccades as well as pupil diameter provided a quantifiable assessment of mental workload in clearcut, wind-damaged, and thinning stands. The indicators analyzed showed that the cutting category can significantly affect the level of mental workload and thus fatigue of harvester operators.

## 1. Introduction

The amount of timber harvested globally has increased by 27% since the 1980s and now stands at 3966 million cubic meters per year [1]. Logging is carried out by different technical means. In many central and eastern European countries, petrol chainsaws are still the main tools used for the felling and cross-cutting of trees [2,3]. At the same time, there is a strong increase in the proportion of stands worked with harvesters. It is estimated that in Poland, about 40% of wood is harvested using fully mechanical technology [4]. In northern and western European countries, mechanical timber harvesting has a longer history and is widely used. According to Lundbäck et al. [5], in Scandinavia, 95% of timber is harvested mechanically, while in western European countries such as Germany, France, and Spain, timber produced by harvesters accounts for 75%, 65%, and 60%, respectively.

The spread of mechanical harvesting has undoubtedly contributed to improving safety, reducing accidents, and improving comfort. This is mainly due to the isolation of the worker from the processed wood and atmospheric conditions [6]. Unfortunately, it is not possible to protect an employee completely from all negative factors in the work environment. The use of harvesters has significantly reduced the severity of work accidents (or work-related illness) and the harmful effects of noise and vibration in comparison with chainsaw work but has also resulted in the exposure of operators to new risks, primarily musculoskeletal disorders resulting from prolonged sitting and repetitive work movements [6,7] and an increase in mental stress [8,9].

Mental workload is an important factor that can affect working efficiency. It is very different from the physical workload and the task load. Mental workload focuses on the stress caused by the complexity of the task, while physical workload focuses mainly on the load on the human body [10,11]. Mental workload also refers to the subjective experience of doing work in a certain environment and at a certain time. Task load refers to the external duties and the number of duties to be performed. Different people may experience different levels of mental workload under the same conditions because of differences in personalities, experiences, cognitive abilities, or skills [11].

There are many factors that influence the working environment of harvester operators, including the model, age, and technical condition of the harvester; the terrain and forest conditions; and the duration and schedule of work shifts. All of these factors directly or indirectly affect the operator’s comfort and thus the rate at which fatigue develops and the risk of adverse health effects occurs.

The issue of harvester operators’ workload is the subject of a growing number of studies, which use a variety of research methods. One of the most commonly used is the measurement of heart rate. This technique was used, among others, by [8,12,13] in studies on the workload of forestry machinery operators. In turn, Dvořák et al. [14] conducted a study to determine the workload during simulated operation using a harvester simulator. The work of a harvester operator is comparable to that of an employee using a computer workstation because it is performed in a sitting position with very little muscular involvement [15]. The energy expenditure of the harvester operator varies between 3.1 and 5.6 kJ/min, which, according to the classification of intensity of physical effort, places it in the category of light work [15]. Harvester operation is a partially automated job but is only apparently undemanding. According to Spinelli et al. [16], working in complex environments such as mixed stands requires high concentration, which contributes to a significant mental load. While cutting, the head positioning and determination of cutting direction is regarded as most demanding task, amplified by a need for quick crane movements and danger of damaging remaining trees. Processing requires a good planning of space for different assortments and making important decisions regarding adequate cross-cutting. For both these operations, a good visibility and focus are crucial. Very often, mechanical harvesting is conducted in shifts. Long working shifts combined with high monotony of work and the need for constant focus can generate problems of operator fatigue, tiredness, and decreased concentration [17].

Physiological measures can be successfully used to assess mental workload. A research technique that has only recently begun to be used to assess workers’ mental workload is eye-tracking. It is a modern, complex, and very demanding technique that uses the detection of movements and measurements of a subject’s eyes to analyze various metrics such as gaze direction, fixations, and saccades [18]. Marquart and de Winter [19] reported that pupillometry, or pupil diameter measurement, is also a good indicator of mental workload. Eye-tracking technology has wide applications in many fields, such as medicine, ergonomics and psychology [20,21,22], linguistics and education [23], economics and marketing [24], cartography [25], and marine shipping [26].

Fixations are phenomena where the subject’s gaze focuses (stops) for a short time [27]. According to Cowen et al. [28], the time of fixational gaze retention on the viewed object varies between 0.2 and 0.3 s. Poole and Ball [18] reported that fixation time ranges from 0.07 to 0.42 s, reaching a mean value of 0.22 s. However, Sałaj [29] and Paśko and Mulawka [30] reported that fixations can last from 0.15 to even 1.5 s.

Saccades are rapid eye movements that occur between fixation points [18,27]. These are very small movements of the eyeball occurring between points that cause interest, and their duration ranges from 0.03 to 0.06 s [31].

The analysis of recordings made with an eye-tracker also provides data on changes in pupil diameter. Many researchers report that this parameter may be an indicator of the rate of fatigue progress, as pupil diameter increases with increasing mental strain [32,33,34].

Despite the widespread use of eye-tracking in commercial research, the majority of scientific publications concern studies under controlled laboratory conditions [35,36,37]. When conducting field studies, account must be taken of additional factors not present in laboratory studies that may reduce the quality of measurements, such as head movements and vibration, distant forward looking, and changes in light intensity that affect changes in pupil diameter [38,39].

There is little work on eye-tracking research in forestry, where it is usually necessary to use mobile devices. The first such study was conducted in New Zealand in the late 20th century by Isler et al. [40]. This involved comparing different color varieties of clothing used by forestry workers, in effect contributing to the widespread use of brightly colored, protective clothing.

Scandinavian researchers [39,41] investigated the areas of interest on which the eyes of harvester operators mostly focus. The study showed that operators focused their gaze the most on the harvester head (average 1.2 s/look), followed by certain parts of the forest, such as the crowns. In addition, different proportions of focus time on various areas of interest were found during the performance of different operations, such as head positioning, felling and processing, etc. Differences have also been demonstrated for different categories of cutting work, such as early thinning, late thinning, and clearcutting [39].

According to Häggström et al. [42], knowledge about operators’ gaze directions can provide valuable information on the perception of specific areas of interest and facilitate the learning of harvester operation. Szewczyk and Sowa [43] conducted an eye-tracking study on the temporal variability of gaze focusing between new and experienced harvester operators. In a subsequent study, Szewczyk et al. compared the performance of harvester operators working in difficult and easy terrain [44] and in wind-damaged stands [45].

The aim of this study is to determine the effect of cutting category on the level of mental workload of operators during logging with a harvester. For this purpose, various saccade and pupil dilation metrics were analyzed. The results may contribute to a better understanding of the work characteristics of the harvester operator, which in the future can be used in the organization of work, e.g., plan the length of work shifts in different logging categories.

## 2. Materials and Methods

### 2.1. Selection of Operators

Operators with a minimum of one year of experience were included in the study. Prior to the start of the study, an interview was conducted with each operator to obtain information regarding age, weight, height, education, and health status. The operators selected had no visual impairments and did not use corrective glasses or contact lenses. Twenty-four experienced operators participated in the study, six for each cutting variant. According to eye-tracking research methodology, this is a sufficient sample to perform a qualitative work analysis [36,44]. All participating operators were informed of the aim of the study and gave their consent to participate in the study.

### 2.2. Machines Involved in the Research

The machines that were used for the study were the ones that the studied operators worked on every day. These included machines from leading manufacturers, such as John Deere, Komatsu, Valmet, Ponsse, and Rottne. On areas with windbreaks, the machines used were: John Deere 1170 E and 1270 E and Komatsu 911 and 931 XT. In clear-cut areas, the following machines were used day and night: John Deere 1070 D and 1270 G, Ponsse Ergo, Rottne H11, and Komatsu 931 XT, whereas in late thinning, Valmet 901 TX, Komatsu 911, John Deere 1270 E and 1270 G, and Ponsse Ergo were used.

### 2.3. Study Areas

Research was conducted in three different categories of timber harvesting: in windbreak areas following the August 2017 hurricane, in clearcuts, and in late thinning. For clearcuts, timber harvesting was conducted during the day and at night. The study was conducted during the summer months. The age of stands in which the surveys were conducted averaged 50 years (27–91) for windbreak areas, 91 years (61–122) for day-shift clearcuts, 103 years (83–133) for night-shift clearcuts, and 70 years (50–108) for late thinning. The study areas were located in lowland areas without significant slopes, with Scots pine (Pinus sylvestris L.) as the dominant species. The average share of Scots pine was 88% (50–100) in windbreak plots, 97% (80–100) in day-shift clearcuts, 98% (90–100) in night-shift clearcuts, and 98% (90–100) in late thinning. Average stand volume was 255 m^3^/ha (213–305) in wind-damaged areas, 367 m^3^/ha (327–466) in day-shift clearcuts, 323 m^3^/ha (217–405) in night-shift clearcuts, and 383 m^3^/ha (288–452) in late thinning.

### 2.4. Study Procedure

The Tobii Pro Glasses 2 mobile eye-tracker was used in this research. During the working shift, 30 min recordings were made four times at one-and-a-half-hour intervals. The first recording started at the beginning of the shift, and the last recording was taken after approximately six hours of continuous work. Taking into account the time spent on setting up the measuring equipment, troubleshooting the machine, and rest breaks, the working shifts, both day and night, amounted to about 8 h. Each of the recordings was subjected to computer analysis in Tobii Pro Lab (x64) version 1.102 (Tobii Technology, Danderyd, Sweden) using the Tobii I-VT attention gaze filter. During the analysis of the recordings, time periods in which the operator performed specific activities (times of interest) were marked. Two times of interest, corresponding to the main work operations, were designated for analysis:

- Felling, which began when the tree was grasped by the head, through the positioning of the head, until the tree was felled, and processing started, or the tree was released from the head;
- Processing, which was from the moment the feed rollers started rotating, through cross-cutting of the logs, until the top was dropped.

These two operations occur consecutively and comprise most of the work time. Other activities, such as driving, machine operation, troubleshooting, break time, and organizational breaks, were hence omitted from the eye-tracking analysis. The following parameters were obtained to infer the psychophysical condition of the operators during operation:

1. Number and duration of saccades (ms);
2. Frequency of saccades (n/s);
3. Proportion of saccade time (%);
4. Mean pupil diameter during fixations (mm);
5. Mean pupil diameter during saccades (mm).

### 2.5. Statistical Analysis

All statistical analyses were performed in the R environment, version 3.6.1 (R Core Team 2019), using R-Studio (version: 1.2.1335). A Lilliefors test was performed to determine the normality of the distribution, due to the large number of observations. Due to the non-normality of the data distribution, differences between variants were tested using the Kruskal–Wallis test, and when differences were found, Dunn’s post hoc test with Bonferroni correction was performed. All tests were performed at a significance level of α = 0.05.

## 3. Results

### 3.1. Duration of Saccades

The highest number of saccades for both felling and processing operations was recorded during clearcutting, while the lowest number was recorded in late thinning (Table 1). In both operations, the highest mean duration of saccades was found in late thinning and the lowest in nighttime clearcutting. The duration of saccades during processing differed significantly between all logging variants (χ^2^(3) = 1148.44, *p* < 0.0001), whereas for felling, statistically significant differences did not occur only between windbreaks and daylight clearcuts (χ^2^(3) = 1232.2, *p* < 0.0001) (Table 1).

### 3.2. Frequency of Saccades

Logging areas produced the highest frequency of saccades during felling during both daytime and nighttime operations (Table 2). The same relationship was observed for the processing operation. For felling activities, statistically significant differences in saccade frequency were found only between the following pairs of categories: logging and thinning areas, nighttime and thinning areas, and disaster and logging areas (χ^2^(3) = 17.3, *p* = 0.00061). For processing, statistically significant differences were observed between thinning and all other cutting categories (χ^2^(3) = 40.70, *p* < 0.0001) (Table 2).

### 3.3. Proportion of Saccade Time

During felling operations, the lowest mean values of proportional saccade time occurred during windbreak removal, slightly higher values during thinning, and the highest values during both clearcut felling variants (Table 3). During processing, a similar pattern was observed as for felling except for windbreaks and late thinning. The lowest mean saccade time proportions were observed in late thinning; the values were more than 10% higher in wind-damaged areas and more than 15% higher during clearcuts. Statistically significant differences in the proportion of saccades during the felling operation were identified only between windbreak and night clearcut areas (χ^2^(3) = 13.20, *p* = 0.0042).

### 3.4. Pupil Diameter

#### 3.4.1. Fixations

More than ten thousand changes in the pupil diameter during fixations were recorded for felling operations in all logging categories (Table 4). The smallest mean pupil diameters were found during harvesting in windbreak areas; slightly higher values were recorded in thinning and clearcut areas during the day. During night work, pupil diameters were significantly higher, exceeding 5 mm. Statistically significant differences were confirmed between all pairs of logging categories (χ^2^(3) = 27,462.56, *p* < 0.0001). The number of changes in pupil diameter was much higher, at approximately 22,000, during the processing operation (Table 4). Again, the largest average pupil diameters were recorded during nighttime logging and the smallest during work in damaged areas. As with felling, statistically significant differences were found between all pairs of cutting categories tested (χ^2^(3) = 45,982.49, *p* < 0.0001).

#### 3.4.2. Saccades

More frequent changes in pupil diameter were recorded during saccades than during fixations, especially for processing (Table 5). During felling, the highest frequency of changes in pupil diameter occurred in clearcut areas. The largest mean pupil diameter values during this operation—above 5.5 mm—were recorded in nighttime clearcut areas. Mean pupil diameter values during processing ranged from 2.89 to 5.52 mm, with the smallest values occurring in windbreak areas and the largest values during nighttime work (Table 5). There were significant differences in pupil diameter during saccades between all pairs of logging categories in both felling (χ^2^(3) = 56,389.99, *p* < 0.0001) and processing of felled trees (χ^2^(3) = 174,923.72, *p* < 0.0001).

## 4. Discussion

The main parameters obtained from the eye-tracking analysis were the number and duration of saccades (Table 1). The results obtained for the average duration of saccades during tree-felling operations are close to the lower limits of the range reported by other researchers. In three logging categories (windbreak areas and day-shift and night-shift clearcuts), similar average saccade times of about 33 ms were recorded during felling, while in thinning, the value was about 39 ms. Values obtained for this operation by Szewczyk et al. [45] were around 41 ms in windbreak areas and around 50 ms in control stands–clear cuts. For the processing operations, the lowest value was recorded in nighttime clearcut areas (33.5 ms) and slightly higher values in daytime clearcut (33.8 ms) areas and windbreaks (34.8 ms). As in the case of felling operations, the highest mean saccade duration was found in thinning areas (36 ms) although it was nearly 3 ms shorter than while felling. For processing, durations showed by Szewczyk et al. were 39 ms in windbreaks and 43 ms in clearcuts. According to Szewczyk et al. [45], a shorter average saccade time during operations in wind-damaged areas compared with normal areas confirms that shorter saccade times indicate a higher workload and a faster rate of fatigue development. This is consistent with the study of May et al. [46], who reported that the duration of saccades decreased as task complexity increased, indicating higher mental workload.

The highest number of saccades was found in nighttime and daytime clearcutting areas. Szewczyk et al. [44] showed that the number of saccades increased during work in difficult and steep terrain. This correlation is also confirmed by Tokuda [47], who reported a higher number of saccadic movements during increased mental strain. The higher number of saccades in clearcutting areas during the day and at night may indicate a higher level of mental strain resulting from the need to undercut trees several times, which reduced work efficiency and increased the perceived risk of head damage or a tree falling over onto the machine. When large-diameter trees are felled, the head must be properly positioned on the trunk, and felling is often preceded by several undercuts. In addition, operators had problems with limited visibility while working at night. During felling, they often looked at the trunk and various elements of the head, which may explain the higher number of saccades. Working under increased strain reduces reaction time and causes rapid shifts of gaze to other parts of the visual scene, as reported by Szewczyk et al. [45].

A higher frequency of saccades was found during processing operations than during felling. In the night-shift clearcutting areas, the frequency of saccades exceeded 10 per second. The frequency was not much lower in the day-shift clearcutting and windbreak areas. During the course of work in these areas, several assortments were harvested, forcing operators to group the logs into several packages. This was frequently hampered by inadequate visibility during night-time operations and the associated need for increased searching of the visual scene. In the windbreak areas, operators struggled with difficult terrain conditions and inadequate working space, due to the large number of rootballs left behind by fallen trees, which hampered the free movement of the machine and correct distribution of the logs. Again, this impacted the amount of time spent searching the visual scene.

During felling, the highest proportion of saccade time was recorded in the night-time and daytime clearcutting areas, where saccades accounted for 16% of the duration of the activity. Similarly, during processing, the highest proportion of saccades occurred in night and day clearcut areas, exceeding 30% of the duration. The lower proportion of saccade time during felling may result from the fact that during this activity the operator’s eyes are focused on the harvester head most of the time. In contrast, the higher saccade time during the processing operation may indicate increased searching of the visual scene. This is because during processing, the operator directs his gaze at more elements of the visual scene, such as the timber, the harvester head and the machine’s surroundings, in order to select the location of the processed assortment. However, one should be cautious in drawing this type of conclusion, as the result may have been indirectly influenced by the overall timing of these activities.

The study also attempted to analyze pupil diameter during fixation and saccades in the course of tree-felling and wood-processing operations. A factor that affects the level of mental strain and the rate at which fatigue develops is work stress. The body’s response to a stressful situation includes an increase in many physiological parameters, including heart rate and blood pressure. Yamanaka and Kawakami [48] reported that there is a correlation between pupil diameter and blood pressure, indicating that when a subject is working under pressure, pupil diameter increases.

In the present study, during both fixation and saccades, slightly higher mean pupil diameters were recorded during felling in all logging variants except late thinning. This indicates that during felling, operators experience higher stress and undergo higher mental strain. This correlation was not confirmed during working in thinning stands. In this case, the larger pupil diameters observed during processing may have been due to the stress of insufficient work space in the stand and the risk of tree damage during movement of the harvester head with a fallen tree. The results confirm the well-known phenomenon whereby pupil diameters become larger with increasing mental strain [32,33,34,47]. However, the recorded differences were rather small in all cases. Moreover, in addition to mental workload, light conditions can also have a significant effect on pupil diameter, making this parameter of limited use in assessing mental workload, especially at night or during long shifts when light conditions change.

Changes in work environment of harvester operator (light conditions, forest types, machine models) are factors that limit the usability of eye-tracking in forestry. Furthermore, there are no guidelines to eye-tracking measurements in demanding forest conditions. Other disadvantages are the need to manipulate in large databases and complicated interpretation of the results. Although being portable, the equipment is rather fragile, and the whole method seems more suitable to stationary research in a controlled environment. Nevertheless, this method gives a number of various metrics to analyze, and using machine learning methods for analysis looks promising. Another interesting idea would be conducting similar eye-tracking research in a harvester simulator environment, as this will enable to control the external conditions.

## 5. Conclusions

An eye-tracking analysis of the work of harvester operators allowed us to determine the level of mental strain during logging in different types of stands. Comparison of the number, duration, incidence, and proportional time of saccades as well as pupil diameter during fixation and saccades provided a quantifiable assessment of mental workload during work in clearcut, wind-damaged, and thinning stands. The indicators analyzed showed that the type of stand in which work is taking place can significantly affect the level of mental strain and thus fatigue of harvester operators. The highest levels of mental workload were found in the clearcut areas during daytime and nighttime operations. It should be emphasized that night work did not differ significantly from daytime work in terms of load.

This study compared two technological operations: felling and tree processing. The results indicated higher slightly mental strain during felling operations. This may be due to stress associated with the higher risk of damage to the machine or remaining trees during this operation. Measurements were also made of operators’ pupil diameters during work. This parameter can be helpful in assessing operator workload, but one must be cautious when interpreting such results because other factors, most notably light intensity, can also influence operators’ pupil diameters. The results obtained indicate in which categories of cutting the operators’ work was most burdensome and may be used in the organization of timber harvesting, for example, for rational planning of the lengths of working shifts.

## Figures and Tables

**Table 1:** Comparison of saccades times between logging variants.

Logging Variant	Number of Saccades	Min (ms)	Max (ms)	Average (ms)	SD
Felling					
Windbreaks	22,176	20	240	33.8 a	21.5
Clearcuts, day	30,327	20	220	33.7 a	20.4
Clearcuts, night	29,542	20	240	33.1 b	21.4
Late thinning	20,221	20	320	38.7 c	23.6
Processing					
Windbreaks	74,997	20	260	34.8 a	21.2
Clearcuts, day	85,220	20	320	33.8 b	20.0
Clearcuts, night	98,610	20	300	33.5 c	21.7
Late thinning	46,058	20	220	36.0 d	20.6

Different letters in the rows indicate significant differences by Kruskal–Wallis test at α = 0.05.

**Table 2:** Comparison of saccade frequency between logging variants.

Logging Variant	Min (n/s)	Max (n/s)	Average (n/s)	SD
Felling				
Windbreaks	0.78	6.44	3.40 ac	1.50
Clearcuts, day	2.16	8.56	4.61 ab	1.78
Clearcuts, night	2.32	8.75	4.82 b	1.50
Late thinning	1.69	5.49	3.26 c	1.02
Processing				
Windbreaks	3.16	14.80	7.47 a	2.85
Clearcuts, day	3.20	18.80	8.96 a	4.25
Clearcuts, night	5.47	16.30	10.10 a	3.55
Late thinning	1.94	7.28	4.09 b	1.39

Different letters in the rows indicate significant differences by Kruskal–Wallis test at α = 0.05.

**Table 3:** Comparison of the proportion of saccades between logging variants.

Logging Variant	Min (ms)	Max (ms)	Average (ms)	SD
Felling				
Windbreaks	3.10	21.60	11.50 a	5.00
Clearcuts, day	7.50	26.60	15.50 ab	4.90
Clearcuts, night	7.80	29.70	16.00 b	4.90
Late thinning	6.80	18.60	12.50 ab	3.90
Processing				
Windbreaks	11.90	52.50	26.10 a	10.00
Clearcuts, day	11.30	61.20	30.20 a	13.40
Clearcuts, night	18.30	53.80	33.90 a	11.10
Late thinning	6.90	22.70	14.70 b	4.30

Different letters in the rows indicate significant differences by Kruskal–Wallis test at α = 0.05.

**Table 4:** Comparison of pupil diameter at fixation time between logging variants.

Logging Variant	Number of Measurements	Min (mm)	Max (mm)	Average (mm)	SD
Felling					
Windbreaks	12,129	1.81	5.42	2.84 a	0.57
Clearcuts, day	14,028	1.94	8.13	3.36 b	0.90
Clearcuts, night	13,095	2.65	7.91	5.50 c	0.90
Late thinning	11,670	1.84	5.80	3.08 d	0.58
Processing					
Windbreaks	22,400	1.86	5.31	2.83 a	0.54
Clearcuts, day	21,998	1,97	6.80	3.24 b	0.81
Clearcuts, night	21,403	2.78	7.80	5.34 c	0.94
Late thinning	22,707	2.06	5.58	3.09 d	0.56

Different letters in the rows indicate significant differences by Kruskal–Wallis test at α = 0.05.

**Table 5:** Comparison of pupil diameter at saccades time between logging variants.

Logging Variant	Number of Measurements	Min (mm)	Max (mm)	Average (mm)	SD
Felling					
Windbreaks	22,176	1.63	6.95	2.91 a	0.62
Clearcuts, day	30,325	1.88	8.97	3.56 b	1.03
Clearcuts, night	29,542	2.62	8.21	5.57 c	0.91
Late thinning	20,221	1.87	8.57	3.13 d	0.63
Processing					
Windbreaks	74,997	1.60	7.58	2.89 a	0.59
Clearcuts, day	85,220	1.65	10.4	3.49 b	0.99
Clearcuts, night	98,610	2.37	9.40	5.52 c	0.97
Late thinning	46,058	1.95	7.04	3.17 d	0.64

Different letters in the rows indicate significant differences by Kruskal–Wallis test at α = 0.05.

## Data Availability

Not applicable.
